# Increasing Adolescent HIV Prevalence in Eastern Zimbabwe – Evidence of Long-Term Survivors of Mother-to-Child Transmission?

**DOI:** 10.1371/journal.pone.0070447

**Published:** 2013-08-07

**Authors:** Jeffrey W. Eaton, Geoffrey P. Garnett, Felicia R. Takavarasha, Peter R. Mason, Laura Robertson, Christina M. Schumacher, Constance A. Nyamukapa, Simon Gregson

**Affiliations:** 1 Department of Infectious Disease Epidemiology, Imperial College London, London, United Kingdom; 2 Bill & Melinda Gates Foundation, Seattle, Washington, United States of America; 3 Biomedical Research and Training Institute, Harare, Zimbabwe; 4 Department of Pediatrics, Johns Hopkins University School of Medicine, Baltimore, Maryland, United States of America; National HIV and Retrovirology Laboratories, Canada

## Abstract

Recent data from the Manicaland HIV/STD Prevention Project, a general-population open HIV cohort study, suggested that between 2004 and 2007 HIV prevalence amongst males aged 15–17 years in eastern Zimbabwe increased from 1.20% to 2.23%, and in females remained unchanged at 2.23% to 2.39%, while prevalence continued to decline in the rest of the adult population. We assess whether the more likely source of the increase in adolescent HIV prevalence is recent sexual HIV acquisition, or the aging of long-term survivors of perinatal HIV acquisition that occurred during the early growth of the epidemic. Using data collected between August 2006 and November 2008, we investigated associations between adolescent HIV and (1) maternal orphanhood and maternal HIV status, (2) reported sexual behaviour, and (3) reporting recurring sickness or chronic illness, suggesting infected adolescents might be in a late stage of HIV infection. HIV-infected adolescent males were more likely to be maternal orphans (RR = 2.97, *p*<0.001) and both HIV-infected adolescent males and females were more likely to be maternal orphans or have an HIV-infected mother (male RR = 1.83, *p*<0.001; female RR = 16.6, p<0.001). None of 22 HIV-infected adolescent males and only three of 23 HIV-infected females reported ever having had sex. HIV-infected adolescents were 60% more likely to report illness than HIV-infected young adults. Taken together, all three hypotheses suggest that recent increases in adolescent HIV prevalence in eastern Zimbabwe are more likely attributable to long-term survival of mother-to-child transmission rather than increases in risky sexual behaviour. HIV prevalence in adolescents and young adults cannot be used as a surrogate for recent HIV incidence, and health systems should prepare for increasing numbers of long-term infected adolescents.

## Introduction

**Figure 1:**
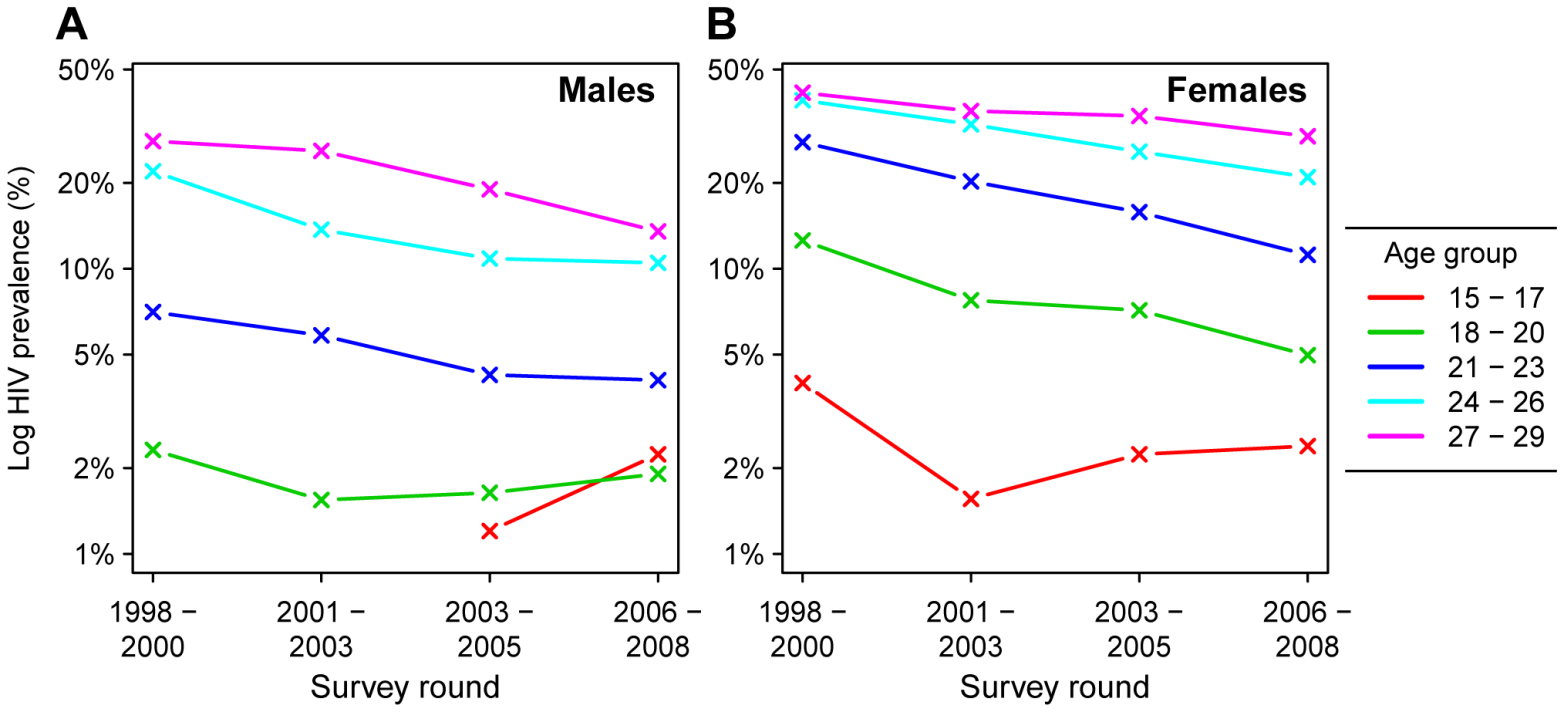
HIV prevalence in adolescents and young adults in the Manicaland HIV/STD Prevention Project. Age-specific HIV prevalence for males (panel A) and females (panel B) by 3-year age groups. Prevalence is represented on the logarithmic scale to illustrate trend at low prevalence levels.

Recent evidence suggests progress in reducing HIV prevalence amongst 15–24 year-olds in several of the most severely affected countries in sub-Saharan Africa [1]. HIV prevalence amongst this group has received special attention because preventing HIV in young adults is crucial for realizing an ‘AIDS-free generation’, and because trends in HIV prevalence amongst young people have often been used as a surrogate for HIV incidence in the absence of direct incidence measures [1], [2]. The Manicaland HIV/STD Prevention Project in rural eastern Zimbabwe was one of the earliest studies to document declining HIV prevalence in southern Africa, and has demonstrated the role of reductions in casual sexual partnerships and delaying sexual debut for reducing the incidence of HIV [3]–[5]. Given this previous evidence of young people’s leading role in reducing HIV incidence in Zimbabwe, and the continuing decline in HIV prevalence in the general adult population, we were surprised by a trend towards increasing HIV prevalence amongst adolescents (aged 15–17 years) in the most recent available data (Figure 1). In 15–17 year-old male study participants, HIV prevalence increased from 1.2% to 2.2% between 2004 and 2007 (*P* = 0.09), and in 15–17 year-old females, prevalence was 2.4% in the most recent round, similar to 2.2% in the previous round (*P* = 0.89). Meanwhile, HIV prevalence declined in male and female young adults (aged 18–29 years).

The uptick in adolescent HIV prevalence could be explained by adoption of risky sexual behaviour by the most recent cohort of adolescents. However, recent clinical reports in Zimbabwe have suggested that a proportion of perinatally infected infants may survive longer than previously believed [6]–[8]. This suggests an alternate hypothesis that some recently detected adolescent HIV infections could be long-term survivors of mother-to-child transmissions (MTCT) that occurred in the early 1990s, the period during which HIV prevalence grew exponentially in pregnant women in Zimbabwe [9]. The observed trend in HIV prevalence among 15–17 year-olds in the Manicaland cohort is temporally consistent with an increasing prevalence of male survivors of MTCT that would be anticipated based on the timing of the epidemic in Zimbabwe, and an increase in adolescent female survivors of MTCT at the same time as a decrease in sexually acquired HIV in adolescent females (Figure S1), although the observed prevalence among 15–17 year-olds in the Manicaland is higher than the proportion of 15–17 year-olds who would be expected to be long-term survivors of MTCT based on Zimbabwe national HIV estimates created using the Spectrum software [10], [11] and survival estimates by Ferrand and colleagues [12] (Figure S1).

The Manicaland HIV cohort only enrolls individuals from the age of 15, so it is impossible to directly observe the source of infection for HIV-infected adolescents. This study examines correlates of perinatal and sexual HIV acquisition and infers the most likely source of the recent uptick in adolescent HIV prevalence in rural Zimbabwe.

## Materials and Methods

### Study Population

The Manicaland HIV/STD Prevention Project is a population-based open cohort in eastern Zimbabwe. The study population consists of 12 geographically distinct communities in Manicaland province (four subsistence-farming areas, two roadside trading settlements, four large-scale agricultural estates, and two rural commercial centres). Four survey rounds have been completed since the establishment of the cohort in 1998. In each survey round, all residents of the study area are enumerated in a household-based census, and eligible adults are invited to join the study. In the most recent survey round, collected between August 2006 and November 2008, all adults aged 15–54 years in a randomly-selected two-thirds of households were eligible to participate. The total population of the cohort sites during the enumeration was 45,124. A total of 14,773 were eligible for the study and 12,166 participated. This study relies principally on 1,948 participants aged 15 to 17 years at the time of completing the survey.

Survey participants completed an approximately one hour-long interview covering education and employment, marital and reproductive history, sexual behaviour, health and health-seeking behaviour, and knowledge and attitudes about HIV. Maternal survival was self-reported by respondents in this survey. If the mother was alive and lived within the study communities, an effort was made by the fieldworker and respondent to identify the mother’s household and line number. If the mother also participated in the most recent study round, we used this to link the respondent and mother’s HIV status. Sensitive sexual behaviour information was reported using the *informal confidential voting interview* (ICVI) method to ensure anonymity and minimize social-desirability bias [13]. Dried blood spots were taken and tested for HIV in an offsite laboratory using the COMBAIDS-RS HIV 1+2 Immunodot Assay (Span Diagnostics, India) and confirmed using Vironostika HIV Uni-form II Plus O (Biomérieux, France).

### Ethics Statement

All respondents (all aged 15 years and older) provided written informed consent at each survey round prior to completing the survey and providing a blood sample. For respondents under age 18 years, written informed consent was also provided by the parent/guardian. Ethical approval for the Manicaland HIV/STD Prevention Project was provided by the Medical Research Council of Zimbabwe and St. Mary’s Local Research Ethics Committee, London.

### Hypotheses

We tested the hypothesis that the increase in adolescent HIV prevalence is attributable to long-term survivors of perinatal HIV transmission. We used individual and household survey data to investigate theoretical correlates of perinatal and sexual HIV acquisition, including maternal survival and HIV infection, sexual behaviour, and chronic illness.

The plausibility of adolescent HIV infections resulting from MTCT relies on mothers of infected adolescents having been infected at the time of birth, 15 to 17 years before participation in the study. Thus, if the source of recent adolescent infections is MTCT, then we would expect (1) that a large proportion of mothers of HIV-infected adolescents are either deceased or HIV-infected, and (2) that mothers of HIV-infected adolescents are more likely to be deceased or HIV-infected than mothers of HIV-uninfected adolescents.

Second, we investigated the association between sexual behaviour and adolescent HIV infection. If the increase in adolescent HIV prevalence is the result of increased sexual acquisition of HIV, then (1) longitudinally, we would expect the increase in adolescent HIV prevalence in the most recent cohort to coincide with a population level increase in sexual risk behaviour amongst adolescents, and (2) at the individual level, there should be a correlation between sexual activity and being HIV-infected.

Finally, if adolescent HIV infections were acquired perinatally, then surviving adolescents would be in late-stage infection and be likely to be experiencing AIDS-related illness associated with lower CD4 count [14]. They should be more likely to be experiencing AIDS-related illness than HIV-infected young adults (aged 18 to 23 years and 24 to 29 years), who were more recently sexually infected. Alternatively, adolescents with recently sexually acquired HIV infection would be less likely to be experiencing AIDS-related symptoms than HIV-infected young adults.

### Statistical Analyses

For adolescents (age 15 to 17 years at the time of survey collection), associations between demographic characteristics and HIV infection were tested for using Fisher’s exact test. For all young adults (age 15 to 29 years), associations were tested for using logistic regression, adjusting for age in three-year age groups.

Maternal status (deceased, HIV-infected, HIV-uninfected, or alive with unknown HIV status) is reported for adolescents by sex and adolescent HIV status. The relative risks of being a maternal orphan and of being an orphan or having a surviving HIV-infected mother are reported for each sex. Statistical significance was evaluated using a one-sided Fisher’s exact test associated with the hypothesis that HIV-infected adolescents are more likely to be orphaned or have HIV-infected mothers.

To investigate longitudinal trends in adolescent sexual behaviour, changes between successive survey rounds in the proportion of adolescent males and females who report ever having had sex and condom usage at last sexual intercourse were tested for using the two-sided Fisher’s exact test.

At the individual level, we tested for an association between HIV infection and reporting ever having had sex amongst adolescents. For those who have had sex, we investigated whether there is an association between HIV infection and the number of sexual partners, having older sexual partners, or reporting condom use at last sex. For males, we tested for an association with self-reported circumcision status. Associations between individual sexual risk behaviours and HIV status were tested using one-sided Fisher’s exact test.

For evaluating whether HIV-infected adolescents were more or less likely to report being chronically ill than HIV positive young adults, respondents were asked “In the last few months have you been in good health, experienced recurring, minor illnesses or been seriously ill?” We tested whether HIV-infected adolescents are more likely to report ‘recurring sickness’ or ‘serious illness’ than HIV-infected young adults, and whether there was an association for HIV-uninfected adolescents and young adults, by fitting log-binomial regression models separately for HIV positive and HIV negative respondents to estimate the relative risk of reporting illness as a function of sex and age group:
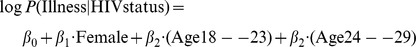



All analyses were conducted using R version 2.14.0 (http://www.r-project.org).

## Results

### Population Characteristics and HIV Status

There were 985 male and 963 female adolescents between ages 15 and 17 years surveyed and tested for HIV during the most recent round of the Manicaland cohort, representing participation of 74.8% and 75.2%, respectively, of those enumerated and eligible to participate. There were 22 HIV-infected adolescent males and 23 HIV-infected females, corresponding to sample HIV prevalences of 2.23% (95% CI 1.40%, 3.36%), and 2.39% (1.84%, 4.02%), respectively (*P* = 0.88). Table 1 shows the association between HIV infection and demographic characteristics amongst adolescents and all study participants aged 15 to 29 years.

**Table 1:** Association between demographic characteristics and HIV infection amongst adolescents and all young adults, year 2006 to 2008.

	Adolescents (age 15–17 years)						All young adults (age 15–29 years)					
	Males			Females			Males			Females		
	n	HIV %	*P*-val*	n	HIV %	*P*-val*	n	HIV %	*P*-val†	n	HIV %	*P*-val†
**Gender**												
Male	985	2.23		–	–	0.881	3204	5.31		–	–	<0.001
Female	–	–		963	2.39		–	–		3889	12.34	
**Age Group**												
15–17							985	2.23	<0.001	963	2.39	<0.001
18–20							734	1.91		824	4.98	
21–23							541	4.07		751	11.19	
24–26							514	10.49		754	20.95	
27–29							429	13.52		597	29.15	
**Community Type**												
Subsist. Farming	452	2.21	0.555	454	1.98	0.425	1196	4.18	0.006	1518	10.21	<0.001
Roadside Trading	257	1.56		206	3.40		704	3.41		777	10.17	
Agricultural Estate	177	2.26		194	1.55		765	5.88		978	12.27	
Commercial Centre	99	4.04		109	3.67		539	9.46		616	20.45	
**Education**												
Primary	124	2.42	0.751	112	2.68	0.743	356	8.15	0.045	708	19.63	<0.001
Secondary	860	2.21		850	2.35		2844	4.96		3174	10.68	
**In school**												
Yes	813	2.34	0.556	719	2.23	0.626						
No	163	1.23		241	2.90							
**Religion**												
Christian	567	2.29	0.632	550	2.36	0.374	1694	4.66	0.742	2014	11.57	0.180
Traditional	20	0.00		4	0.00		70	7.14		38	15.79	
Spiritual	189	1.59		244	1.64		603	4.98		1004	12.85	
Other	140	2.14		142	3.52		433	4.85		687	12.37	
None	63	4.76		16	6.25		387	8.53		119	21.85	
**Marital Status**												
Never married	980	2.24		885	2.60	0.435	2409	2.95	0.001	1547	3.81	<0.001
Married	0			71	0.00		722	12.05		1956	14.52	
Widowed	0			0			4	25.00		72	65.28	
Divorced/Separated	0			2	0.00		51	19.62		281	28.47	
Been tested and												
received result§	9	11.11	0.174	46	6.52	0.085	224	9.38	0.434	1109	12.98	<0.001
Not tested	949	2.00		906	2.10		2909	4.95		2735	12.14	
Self report HIV+	2	1/2		3	3/3		11	27.27		50	92.00	
Self report HIV−	11	0.00		44	0.00		218	8.26		1064	9.02	
Ever taken ART	1			1			1			10		

* *P*-value associated with two-sided Fisher’s exact test.

† *P*-value associated with likelihood ratio test adjusting for age group.

§ “Tested and received result” based on answering more than zero to question “On how many different occasions did you have an HIV test?” and answering ‘yes’ to a follow-up question about the most recent HIV test “Did you collect the test results?”.

Amongst adolescents, none of the demographic characteristics were significantly associated with HIV infection, including sex–females were not more likely to be infected than males. Amongst all young adults, HIV infection was associated with older age, being married or widowed, and living on an agricultural estate. Less education was significantly associated with HIV infection for females but not males. In univariate analysis, women who were not members of any church were more likely to be infected (*P* = 0.04), but the association was not significant after adjusting for age group.

Uptake of HIV testing was low amongst adolescents. Only 0.9% and 4.8% of adolescent males and females, respectively, reported ever being tested for HIV and collecting the test result. Only 1 adolescent male and 1 adolescent female reported having taken drugs to stop HIV from causing AIDS, and only 11 people overall under age 30 reported having taken such drugs.

### Maternal Survival and HIV Infection

Table 2 reports the maternal status of HIV-infected and uninfected adolescents. Fourteen out of 22 (64%) of HIV-infected adolescent males reported being maternal orphans. We were able to link the HIV status for six of eight surviving mothers, and three were HIV positive. HIV-infected adolescent males were 2.97 times more likely to be maternal orphans than uninfected adolescent males, and were 1.83 times more likely to have mothers who were deceased or HIV-infected, excluding mothers who were living with unknown HIV status (both *P*<0.001). For females, seven out of 23 reported being maternal orphans (30%). This made them 1.47 times more likely to be orphans than HIV-uninfected adolescent females, which was not statistically significant (*P* = 0.30). Of the 16 who reported surviving mothers, we were able to link the HIV status for 10. Nine of these were HIV-infected, making HIV-infected females 16.6 times (*P*<0.001) more likely to have deceased or HIV-infected mothers than uninfected female adolescents. Amongst HIV-positive adolescents who had a surviving HIV-positive mother (n = 12), none of the mothers were observed to have seroconverted between previous survey rounds, confirming that maternal HIV infection was acquired at least before enrolment in the cohort.

**Table 2:** Maternal mortality and HIV status by adolescent HIV status.

	Adolescent males*		Adolescent females*	
	HIV +	HIV −	HIV +	HIV −
	(N = 22)	(N = 961)	(N = 23)	(N = 936)
Mother deceased	14 (64%)	206 (21%)	7 (30%)	194 (21%)
Mother alive & HIV positive	3 (14%)	102 (11%)	9 (39%)	90 (10%)
Mother alive & HIV negative	3 (14%)	354 (37%)	1 (4%)	343 (37%)
Mother alive & unknown HIV status	2 (9%)	299 (31%)	6 (26%)	309 (33%)
RR of mother being deceased†	2.97 (*P*<0.001)		1.47 (*P* = 0.297)	
RR of mother deceased or HIV+†,§	1.83 (*P*<0.001)		16.62 (*P*<0.001)	

* There are slightly fewer respondents than in Table 1 due to missing data about maternal survival for 2 male and 4 female respondents (all HIV-negative).

† *P-*value based on one-sided Fisher’s exact test.

§ excludes adolescents with surviving mothers with unknown HIV status.

### Sexual Behaviour

**Figure 2:**
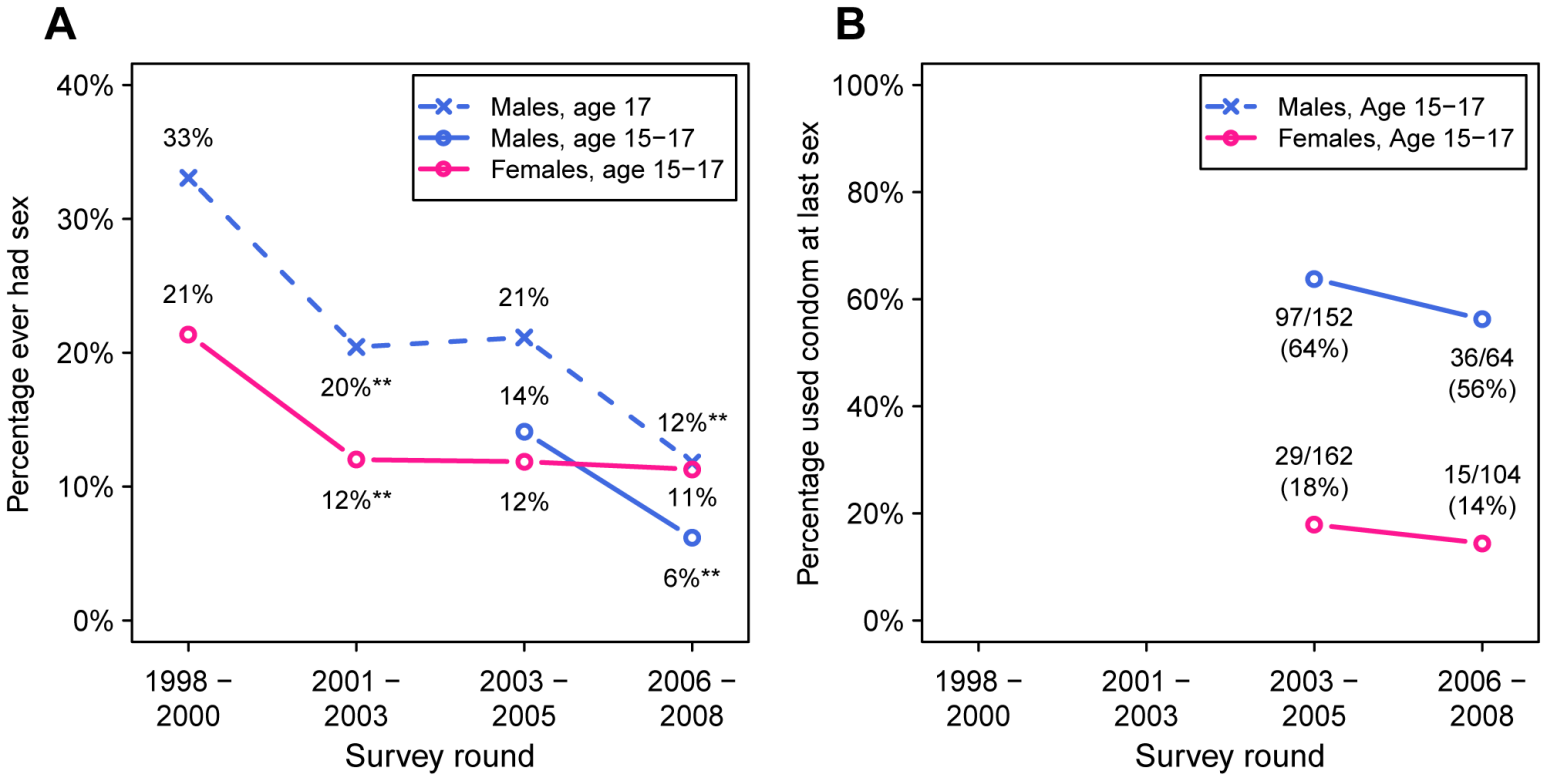
Trends in reported sexual behaviour amongst adolescents age 15 to 17 years. Panel A shows the percentage of adolescents in each cohort who report ever having had sex. Panel B indicates the percentage of respondents who reported using a condom at their last sex, amongst those who have ever had sex. Asterisks denote a statistically significant decline between cohorts (two-sided Fisher’s exact test, all significant at level *P*<0.001). Because males were only enroled from the age of 17 in the first two survey rounds, the dashed blued line shows the trend in the proportion of sexually experienced 17 year-old males, and the solid blue line shows the trend for 15 to 17 year-old males in the two most recent rounds.

Figure 2 shows longitudinal trends in the proportion of adolescents in each survey round reporting ever having had sex (panel A), and the proportion of those reporting condom use at last sex (panel B). During the same time period that HIV prevalence in adolescent males increased from 1.2% to 2.2%, the proportion of adolescent males reporting ever having had sex decreased from 14% to 6% (*P*<0.001). The proportion of adolescent females reporting ever having sex did not change significantly from 12% to 11% (*P* = 0.70). There were small decreases in the proportion of both males and females reporting using a condom at last sex, which were not statistically significant. These estimates are based on small numbers of sexually active adolescents, and changes must be considered in the context of the precipitous decline in sexually-active adolescent males.

At the individual level, none of 22 HIV-infected adolescent males reported ever having sex (Table 3), nullifying investigation of further sexual risk behaviours. Overall, there was not an association between lack of circumcision and HIV infection (RR 0.33, one-sided *P* = 0.97). Three out of 23 HIV-infected adolescent females reported ever having had sex. Among all female adolescents, HIV infection was not associated with ever having had sex (*P* = 0.49), having older sexual partners, having multiple sexual partners (*P* = 0.60), or lack of condom usage at last sex (*P* = 0.73).

**Table 3:** Individual Risk Factors for Adolescent Sexual HIV Acquisition.

	Males				Females			
	N	HIV %	RR	*P*-val†	N	HIV %	RR	*P*-val†
Total	985	2.23			963	2.39	1.07††	0.881††
Never Had Sex	924	2.38	1		854	2.34	1	
Had Sex	61	0.0	0.0	1.0	109	2.75	1.18	0.493
Partner > = 25	6	0.0			8	0.0		
Partner > = 20	1	0.0			34	0.0		
Partner <20	2	0.0			23	0.0		
Unknown Age	52	0.0			44	6.82		
1 lifetime partner	33	0.0			72	2.78	1	
>1 lifetime partner	27	0.0			25	4.00	1.44	0.596
Used condom last sex	33	0.0			15	0.0		
No condom last sex	24	0.0			88	2.27		0.729
Circumcised	31	6.45	1					
Uncircumcised	951	2.10	0.33	0.971				

† *P*-value associated with one-sided Fisher exact test except where noted otherwise.

†† relative risk of female being infected vs. male being infected, two-sided *P*-value.

### Illness

The number of respondents reporting recurring sickness or serious illness is presented in Table S1 by sex, age, and HIV status. Table S2 shows the results of relative risk regression of the influence of age on the likelihood of reporting illness for HIV-infected and uninfected respondents. In both models, females were more likely to report illness; the relative risk was 1.65 (95% CI 1.14, 2.40) amongst HIV-infected respondents and 1.85 (1.59, 2.14) for those HIV-uninfected. For HIV-infected respondents, 18–23 year olds and 24–29 year olds were less likely than 15–17 year olds to report illness, with relative risks of 0.61 (0.37, 1.03) and 0.65 (0.41, 1.03), respectively. This is consistent with the hypothesis that infected adolescents are more likely to be experiencing AIDS-related symptoms. For HIV-uninfected respondents, the relative risk of reporting illness, compared to 15–17 year olds, were 0.87 (0.74, 1.02) and 0.88 (0.74, 1.05) for 18–23 and 24–29 year olds, respectively.

## Discussion

All three correlates of perinatal and sexual HIV transmission that we tested were consistent with the hypothesis that newly detected HIV infections in adolescents are primarily attributable to mother-to-child HIV transmission: mothers of HIV-infected adolescents were much more likely to be deceased or HIV-infected themselves, HIV infection was not associated with sexual risk behaviours in adolescent males or females, and HIV-infected adolescents were more likely to report illness, indicative of being in late-stage HIV infection, than were HIV-infected young adults. To our knowledge, this is the first population-based cohort data to demonstrate that, as generalized HIV epidemics in southern Africa mature, a substantial proportion of HIV infections in older adolescents and young adults may be attributable to long-term survivors of perinatal infections. This supports predictions by others of an increasing prevalence of HIV infection in adolescents due to mother-to-child transmission based on clinical reports [6]–[8], cross-sectional HIV prevalence data [15], and mathematical models [12]. Although this study was done using data primarily from a single round of an ongoing cohort study in a geographically stratified population in Manicaland province, other research has found the epidemic in this population to be largely consistent with trends in the national HIV epidemic [5], and the conclusions of the hypotheses tested here should be applicable across the region. Importantly, these findings from eastern Zimbabwe could be an early warning for other countries in southern Africa with severe HIV epidemics that exploded a few years after that in Zimbabwe.

It would be a misinterpretation of these results to conclude that, because HIV prevalence only increased in adolescent males and remained flat in adolescent females, we only found support for long-term male survivors. While the associations with maternal HIV and sexual behaviour were stronger for males, they were significant for females. To our knowledge, there is not data to suggest that infant males would be more likely to perinatally acquire HIV, or be more likely to be long-term survivors. Instead, in light of the reductions in sexual risk behaviour reported by adolescent females documented here and elsewhere [3]–[5], the constant HIV prevalence in adolescent females could conceal a decline in sexually acquired HIV amongst adolescent females at the same time as the number of long-term survivors increase. HIV incidence in adolescent males has always been relatively low, causing the increase in survivors of earlier infection to be the dominant trend.

The potential source of infection for three adolescent males whose mothers were HIV negative and reported never having had sex remains uncertain. Other non-sexual modes of HIV transmission, such as contaminated medical injections, exposure caring for ill family members, or misreporting of sexual behaviour cannot be excluded for these individuals or other adolescents who are infected but do not report sexually activity. Reporting medical injections in the past three years was not associated with HIV infection for the adolescents in this study, and previous research found that medical injections were not associated with adult HIV incidence in this population [16]. Considering other research suggesting that a proportion of perinatally infected infants may have long survival [12], [17], we believe that this is the most parsimonious explanation for the majority of the adolescent HIV infections. However, determining this with more certainty would increase confidence in projections of the future trend in HIV amongst children and adolescents, inform whether we can expect the prevalence of these infections to follow patterns in antenatal HIV prevalence and the scale-up of antiretroviral based prevention of mother-to-child transmission, and shed light on whether there are other sources of infection that require prevention attention. Where possible, further research should more decisively determine the source of non-sexual HIV infections amongst children and adolescents. Phylogenetic approaches to linking HIV transmissions and estimating the duration of infection may be a way to overcome some of the challenges inherent in designing epidemiological studies to determine the time and source of infections that happened many years in the past [18], [19].

One limitation of this study is that maternal survival, sexual behaviour, and health are self-reported. Underreporting of sexual behaviour could lead to misclassification of sexual transmissions [20]–[22]. However, one indication that we have not erred in interpreting the lack of association between HIV infection and sexual activity as evidence against sexual HIV acquisition is that there was a strong association between adolescent sexual activity and being HIV-infected in previous survey rounds, when adolescent HIV infections were almost surely sexually acquired (Figure S2). If the majority of recently observed adolescent HIV infections actually were sexually acquired, not only would there have had to been a substantial underreporting of sexual activity amongst HIV-infected adolescents to artificially create the lack of association, but this bias would have to increased substantially over the survey rounds.

Maternal survival and identification could also have been misreported, perhaps due to misclassifying adoptive mothers as biological mothers [23]. Among the 24 infected adolescents who reported that their biological mother was alive, household census records indicated that two were maternal orphans (both were females classified as ‘mother alive, unknown HIV status’ in Table 2). Inclusion of these as orphans would strengthen the association between adolescent HIV and maternal orphanhood reported in Table 2. Household census records were discrepant about the orphanhood status of three HIV-infected adolescent males who reported that their mother was deceased.

We questioned whether it was plausible that many mothers who transmitted HIV to infants would survive 15 years without access to antiretroviral therapy. We decided that it was based on data suggesting fairly long survival distributions for women infected in their late teens and twenties [24], [25]. Moreover, the increased risk of HIV acquisition during pregnancy [26], [27], high transmissibility during early HIV infection [28], and the epidemic trajectory make it likely that mothers were early in HIV infection themselves when perinatal transmission occurred [29]. Others studies, before the hypothesized prevalence of long-term survivors of MTCT, have reported significant associations between maternal orphanhood and (presumptively sexually acquired) HIV infection in adolescents [30]–[37], but these associations were mediated by increased sexual risk behaviour among maternal orphans, for which we did not find support.

For each of the individual hypotheses tested, one can conceive of alternative explanations for the observed correlations, but it is compelling that tests of all of the *a priori* hypotheses supported the same conclusion. The shift in the source of infections in adolescents from sexual transmission to survivors of MTCT represents another important epidemiological transition, and has several important implications. First, it strongly affects the interpretation of surveillance data. HIV infections amongst adolescents cannot be presumed to be recent infections, and hence trends in adolescent and young adult HIV prevalence cannot be interpreted as a proxy for recent trends in HIV incidence or indicators of the success or failure of HIV prevention programmes. This reiterates the supreme importance of routinely and accurately measuring HIV incidence. Second, the survival of untreated perinatally infected infants until their late teens or early twenties poses new challenges for HIV prevention because of the potential for onward transmission. Should perinatally-infected adolescents begin unprotected sexual activity, they may be highly infectious due to high viral load associated with late-stage HIV infection. Further research is needed on sexual risk behaviour amongst long-term survivors [38]. Finally, the scope of HIV testing, treatment, and care efforts must urgently be expanded to focus on children and adolescents, including routine HIV testing of children and adolescents presenting for acute primary care and hospital care [6]–[8]. Young people living with HIV often face different needs and challenges than older adults [39], to whom HIV care programmes are typically catered. Provision of HIV testing and early antiretroviral therapy is likely to be integral to the prevention of onward transmission by HIV-infected adolescents.

## Supporting Information

Figure S1 **Comparison of model projection of long-term survivors of perinatal infection with observed adolescent HIV prevalence in Manicaland HIV/STD Prevention Project.**
(DOCX)

Figure S2 **Association between reporting sexual activity and adolescent HIV infection in four survey rounds.**
(DOCX)

Table S1 **Reporting illness by sex, age, and HIV status.**
(DOCX)

Table S2 **Association Between Age and Reporting Recurring or Chronic Illness in the Past Few Months for HIV Positive and HIV Negative Adolescents and Young Adults.**
(DOCX)

Table S3 **Maternal survival and maternal HIV infection for young adults by age, gender, and HIV status.**
(DOCX)

Table S4 **Associations between lifetime sexual partners and HIV infection for young adults.**
(DOCX)

Text S1 **Assumptions and data for model estimate of proportion of long-term survivors of MTCT.**
(DOCX)
