# Supplementary material for: Increasing Adolescent HIV Prevalence in Eastern Zimbabwe – Evidence of Long-Term Survivors of Mother-to-Child Transmission?
Source: PLoS One. 2013 Aug 7;8(8):e70447. doi: 10.1371/journal.pone.0070447 (PMC3737189; doi:10.1371/journal.pone.0070447)
Supplement: Figure S1 — Comparison of model projection of long-term survivors of perinatal infection with observed adolescent HIV prevalence in Manicaland HIV/STD Prevention Project. (DOCX) [file pone.0070447.s001.docx]

Figure S1. Comparison of model based projection of adolescent survivors of mother-to-child transmission with HIV prevalence amongst adolescents participating in the Manicaland HIV/STD Prevention Project. The grey line indicates the estimated national HIV prevalence amongst newborn infants from the official Zimbabwe national estimates created using the Spectrum software and reported in the UNAIDS Global Report 2012 [1,2]. Black circles indicate the estimated percentage of living adolescents aged 15 to 17 years who are survivors of mother-to-child transmission that would be expected if perinatally infected infants survive according to the survival model proposed by Ferrand *et al* [3]. Pink crosses indicate HIV prevalence amongst age 15 to 17 year-old female participants in each of the four rounds of the Manicaland cohort. Blue crosses indicate HIV prevalence amongst male adolescent participants in the Manicaland cohort.
