# Supplementary material for: Increasing Adolescent HIV Prevalence in Eastern Zimbabwe – Evidence of Long-Term Survivors of Mother-to-Child Transmission?
Source: PLoS One. 2013 Aug 7;8(8):e70447. doi: 10.1371/journal.pone.0070447 (PMC3737189; doi:10.1371/journal.pone.0070447)
Supplement: Figure S2 — Association between reporting sexual activity and adolescent HIV infection in four survey rounds. (DOCX) [file pone.0070447.s002.docx]

**Figure S2.** Relative risk of being HIV infected for adolescents age 15 to 17 who report ever having had sex relative to those who report never having had sex in each survey round of the Manicaland HIV/STD Prevention Project. In earlier survey rounds reporting ever having had sex is strongly associated with being HIV positive. During previous survey rounds, adolescent HIV infections were most likely to have been sexually acquired and many survivors of MTCT would not be predicted given the temporal trajectory of the epidemic.
