# Supplementary material for: Increasing Adolescent HIV Prevalence in Eastern Zimbabwe – Evidence of Long-Term Survivors of Mother-to-Child Transmission?
Source: PLoS One. 2013 Aug 7;8(8):e70447. doi: 10.1371/journal.pone.0070447 (PMC3737189; doi:10.1371/journal.pone.0070447)
Supplement: Table S1 — Reporting illness by sex, age, and HIV status. (DOCX) [file pone.0070447.s003.docx]

**Table S1.** Reporting Illness by Sex, Age, and HIV Status.

|  | HIV infected | | HIV uninfected | | RR^†^ | *P*-value |
| --- | --- | --- | --- | --- | --- | --- |
|  | N | Ill (%) | N | Ill (%) |  |  |
| **Male** |  |  |  |  |  |  |
| 15–17 | 22 | 4 (18%) | 963 | 86 (9%) | 2.04 | 0.134 |
| 18–23 | 36 | 4 (11%) | 1239 | 80 (6%) | 1.72 | 0.292 |
| 24–29 | 112 | 20 (18%) | 832 | 70 (8%) | 2.12 | 0.003 |
|  |  |  |  |  |  |  |
| **Female** |  |  |  |  |  |  |
| 15–17 | 22 | 10 (43%) | 940 | 144 (15%) | 2.84 | <0.001 |
| 18–23 | 125 | 31 (25%) | 1450 | 208 (14%) | 1.73 | 0.003 |
| 24–29 | 332 | 83 (25%) | 1019 | 134 (13%) | 1.89 | <0.001 |
