# Supplementary material for: Increasing Adolescent HIV Prevalence in Eastern Zimbabwe – Evidence of Long-Term Survivors of Mother-to-Child Transmission?
Source: PLoS One. 2013 Aug 7;8(8):e70447. doi: 10.1371/journal.pone.0070447 (PMC3737189; doi:10.1371/journal.pone.0070447)
Supplement: Table S2 — Association Between Age and Reporting Recurring or Chronic Illness in the Past Few Months for HIV Positive and HIV Negative Adolescents and Young Adults. (DOCX) [file pone.0070447.s004.docx]

**Table S2.** Association Between Age and Reporting Recurring or Chronic Illness in the Past Few Months for HIV Positive and HIV Negative Adolescents and Young Adults.

|  | **Model: HIV Positive** | | |  | **Model: HIV Negative** | | |
| --- | --- | --- | --- | --- | --- | --- | --- |
|  | RR | 95% CI | *P*-value |  | RR | 95% CI | *P*-value |
| **Intercept:** | 0.24 | 0.15, 0.39 |  |  | 0.09 | 0.07, 0.10 |  |
| **Sex:** |  |  |  |  |  |  |  |
| Male | 1 |  |  |  | 1 |  |  |
| Female | 1.65 | 1.14, 2.40 | 0.008 |  | 1.85 | 1.59, 2.14 | <0.001 |
| **Age group:** |  |  |  |  |  |  |  |
| 15-17 | 1 |  |  |  | 1 |  |  |
| 18-23 | 0.61 | 0.37, 1.03 | 0.064 |  | 0.87 | 0.74, 1.02 | 0.085 |
| 24-29 | 0.65 | 0.41, 1.03 | 0.069 |  | 0.88 | 0.74, 1.05 | 0.171 |
|  |  |  |  |  |  |  |  |
