# Supplementary material for: Increasing Adolescent HIV Prevalence in Eastern Zimbabwe – Evidence of Long-Term Survivors of Mother-to-Child Transmission?
Source: PLoS One. 2013 Aug 7;8(8):e70447. doi: 10.1371/journal.pone.0070447 (PMC3737189; doi:10.1371/journal.pone.0070447)
Supplement: Table S3 — Maternal survival and maternal HIV infection for young adults by age, gender, and HIV status. (DOCX) [file pone.0070447.s005.docx]

**Table S3.** Maternal Survival and Maternal HIV Infection for Young Adults by Age, Sex, and HIV Status.

| Age Group | HIV Status | Mother deceased | Mother alive, HIV+ | Mother alive, HIV- | Mother alive, unknown | RR mother deceased | *P*-value |
| --- | --- | --- | --- | --- | --- | --- | --- |
|  |  |  |  |  |  |  |  |
| **Males** |  |  |  |  |  |  |  |
| Age 15–17 | HIV+ | 14 (64%) | 3 (14%) | 3 (14%) | 2 (9%) | 2.97 | < 0.001 |
|  | HIV- | 206 (21%) | 102 (11%) | 354 (37%) | 299 (31%) |  |  |
| Age 18–23 | HIV+ | 11 (31%) | 2 (6%) | 9 (26%) | 13 (37%) | 1.40 | 0.220 |
|  | HIV- | 277 (22%) | 66 (5%) | 337 (27%) | 555 (45%) |  |  |
| Age 24–29 | HIV+ | 25 (23%) | 1 (1%) | 12 (11%) | 73 (66%) | 0.92 | 0.724 |
|  | HIV- | 202 (24%) | 20 (2%) | 114 (14%) | 491 (59%) |  |  |
|  |  |  |  |  |  |  |  |
| **Females** |  |  |  |  |  |  |  |
| Age 15–17 | HIV+ | 7 (30%) | 9 (39%) | 1 (4%) | 6 (26%) | 1.47 | 0.297 |
|  | HIV- | 194 (21%) | 90 (10%) | 343 (37%) | 309 (33%) |  |  |
| Age 18–23 | HIV+ | 41 (33%) | 3 (2%) | 6 (5%) | 73 (59%) | 1.34 | 0.041 |
|  | HIV- | 358 (25%) | 60 (4%) | 244 (17%) | 778 (54%) |  |  |
| Age 24–29 | HIV+ | 94 (29%) | 7 (2%) | 21 (6%) | 206 (63%) | 1.20 | 0.092 |
|  | HIV- | 242 (24%) | 13 (1%) | 69 (7%) | 686 (68%) |  |  |
