# Supplementary material for: Increasing Adolescent HIV Prevalence in Eastern Zimbabwe – Evidence of Long-Term Survivors of Mother-to-Child Transmission?
Source: PLoS One. 2013 Aug 7;8(8):e70447. doi: 10.1371/journal.pone.0070447 (PMC3737189; doi:10.1371/journal.pone.0070447)
Supplement: Table S4 — Associations between lifetime sexual partners and HIV infection for young adults. (DOCX) [file pone.0070447.s006.docx]

**Table S4.** Associations Between Lifetime Sexual Partners and HIV Infection for Young Adults.

|  | Males | | | | | | | | |  | Females | | | | | | | | |
| --- | --- | --- | --- | --- | --- | --- | --- | --- | --- | --- | --- | --- | --- | --- | --- | --- | --- | --- | --- |
|  | Age 15-17 | | | Age 18-23 | | | Age 24-29 | | |  | Age 15-17 | | | Age 18-23 | | | Age 24-29 | | |
|  | n | HIV % | *P*-val | n | HIV % | *P*-val | n | HIV % | *P*-val |  | n | HIV % | *P*-val | n | HIV % | *P*-val | n | HIV % | *P*-val |
| Lifetime  Partners |  |  |  |  |  |  |  |  |  |  |  |  |  |  |  |  |  |  |  |
| 0 | 869 | 2.19 | 1.00 | 546 | 1.10 | <0.001 | 41 | 0.0 | <0.001 |  | 808 | 2.35 | 0.318 | 422 | 0.95 | <0.001 | 35 | 2.86 | <0.001 |
| 1 | 33 | 0.0 |  | 212 | 1.89 |  | 183 | 5.46 |  |  | 72 | 3.78 |  | 777 | 9.78 |  | 787 | 19.31 |  |
| 2 | 12 | 0.0 |  | 130 | 6.92 |  | 154 | 12.99 |  |  | 15 | 0.0 |  | 209 | 11.48 |  | 307 | 29.32 |  |
| 3-5 | 11 | 0.0 |  | 201 | 5.47 |  | 300 | 12.00 |  |  | 9 | 11.11 |  | 61 | 18.03 |  | 124 | 39.52 |  |
| > 5 | 4 | 0.0 |  | 110 | 2.73 |  | 247 | 18.22 |  |  | 1 | 0.0 |  | 20 | 30.00 |  | 45 | 64.44 |  |
|  |  |  |  |  |  |  |  |  |  |  |  |  |  |  |  |  |  |  |  |
| Circumcised | 31 | 6.45 | 0.151 | 69 | 4.35 | 0.441 | 71 | 16.90 | 0.181 |  |  |  |  |  |  |  |  |  |  |
| Uncircumcised | 951 | 2.10 |  | 1201 | 2.75 |  | 873 | 11.45 |  |  |  |  |  |  |  |  |  |  |  |
