# Supplementary material for: Increasing Adolescent HIV Prevalence in Eastern Zimbabwe – Evidence of Long-Term Survivors of Mother-to-Child Transmission?
Source: PLoS One. 2013 Aug 7;8(8):e70447. doi: 10.1371/journal.pone.0070447 (PMC3737189; doi:10.1371/journal.pone.0070447)
Supplement: Text S1 — Assumptions and data for model estimate of proportion of long-term survivors of MTCT. (DOCX) [file pone.0070447.s007.docx]

**Text S1.** Assumptions and data for model estimate of proportion of long-term survivors of MTCT

Model-based projections of the expected proportion of adolescents, age 15 to 17 years old, in 1999 to 2009 who are survivors of mother-to-child transmission from years (Figure 2) are estimated by combining estimates of the annual number of HIV-infected and total newborns each year from the official Zimbabwe national HIV estimates created using the Spectrum software , published in the UNAIDS Global Report 2012 [1], with the survival function for HIV-infected infants estimated by Ferrand *et al* [3].

The assumed number of HIV-infected number of newborns in each year, denoted by $N^{+}(t)$ and $N(t)$ respectively, are reproduced in Table S6.

**Table S6:** Annual number of total and HIV-infected births in Zimbabwe, estimates from Zimbabawe national estimates reported in the UNAIDS Global Report 2012 [3]

| **Year** | **HIV infected births (**$N^{+}(t)$**)** | **Total Births**  **(**$N(t)$**)** | **Newborn HIV Prevalence** |
| --- | --- | --- | --- |
| 1981 | 280 | 339,466 | 0.1% |
| 1982 | 567 | 349,898 | 0.2% |
| 1983 | 1,079 | 358,116 | 0.3% |
| 1984 | 1,921 | 363,206 | 0.5% |
| 1985 | 3,197 | 367,182 | 0.9% |
| 1986 | 4,973 | 370,959 | 1.3% |
| 1987 | 7,293 | 374,378 | 1.9% |
| 1988 | 10,184 | 378,344 | 2.7% |
| 1989 | 13,579 | 382,592 | 3.5% |
| 1990 | 17,253 | 384,828 | 4.5% |
| 1991 | 21,020 | 385,636 | 5.5% |
| 1992 | 24,858 | 385,218 | 6.5% |
| 1993 | 29,054 | 384,700 | 7.6% |
| 1994 | 33,148 | 384,489 | 8.6% |
| 1995 | 36,317 | 383,538 | 9.5% |
| 1996 | 38,712 | 381,768 | 10.1% |
| 1997 | 40,283 | 379,282 | 10.6% |
| 1998 | 41,160 | 378,388 | 10.9% |
| 1999 | 41,573 | 381,576 | 10.9% |
| 2000 | 41,071 | 383,462 | 10.7% |
| 2001 | 39,827 | 384,000 | 10.4% |
| 2002 | 38,017 | 383,446 | 9.9% |
| 2003 | 35,726 | 383,412 | 9.3% |
| 2004 | 32,710 | 384,366 | 8.5% |
| 2005 | 29,887 | 385,535 | 7.8% |
| 2006 | 27,893 | 396,060 | 7.0% |
| 2007 | 25,861 | 406,787 | 6.4% |

The probability of surviving to age $a$ for HIV-infected infants is given by the function

$S^{+}\left( a \right)=\gamma*e^{-\beta*a}+\left( 1-\gamma\right)*2^{-\left( \frac{a}{\mu} \right)^{\eta}}$,

where $\gamma=0.57$, $\beta=1.52$, $\mu=16$, and $\eta=2.7$. Survival for HIV-uninfected newborns, denoted by $S^{-}\left( a \right)$, is based age-specific mortality rates from the World Health Organization Global Health Observatory [4] life table for Zimbabwe in the year 1990, before a high prevalence of HIV in children. Mortality rates for HIV-uninfected survivors are in Table S7.

**Table S7:** Age-specific mortality rates for HIV-uninfected newborns, based on the WHO model life table for Zimbabwe in year 1990

| **Age (years)** | **Annual mortality rate** |
| --- | --- |
| < 1 | 0.05450 |
| 1 – 4 | 0.00717 |
| 5 – 9 | 0.00195 |
| 10 – 14 | 0.00152 |
| 15 – 19 | 0.00257 |

Combining these functions, the proportion of adolescents who are survivors of MTCT in year $y$ are estimated according to the function

$$P\left( y \right)=\frac{\sum_{a=15}^{17} N^{+}\left( y-a \right)\cdot S^{+}\left( a+0.5 \right)}{\sum_{a=15}^{17} N^{+}\left( y-a \right)\cdot S^{+}\left( a+0.5 \right)+\left[ N\left( y-a \right)-N^{+}\left( y-a \right) \right]\cdot S^{-}\left( a+0.5 \right)} .$$

**References**

1. Joint United Nations Programme on HIV/AIDS (UNAIDS) (2012) Global report: UNAIDS report on the global AIDS epidemic 2012: 103. Available: http://www.unaids.org/en/media/unaids/contentassets/documents/epidemiology/2012/gr2012/20121120_UNAIDS_Global_Report_2012_with_annexes_en.pdf. Accessed 12 February 2013.

2. Stover J, Johnson P, Hallett T, Marston M, Becquet R, et al. (2010) The Spectrum projection package: improvements in estimating incidence by age and sex, mother-to-child transmission, HIV progression in children and double orphans. *Sex Transm Infect* 86 Suppl 2: ii16–21.

3. Ferrand RA, Corbett EL, Wood R, Hargrove J, Ndhlovu CE, et al. (2009) AIDS among older children and adolescents in Southern Africa: projecting the time course and magnitude of the epidemic. *AIDS* 23: 2039–2046.

4. World Health Organization. (2013) Global Health Observatory Data Repository, Life expectancy: Life tables Zimbabwe. Available: <http://apps.who.int/gho/data/view.main.61860>. Accessed 27 May 2013.
